# Current News

**Published:** 1903-05

**Authors:** 


### Current News.

### NEW JERSEY STATE BOARD OF REGISTRATION AND EXAMINATION IN DENTISTRY.

The New Jersey State Board of Registration and Examination in Dentistry will hold its semiannual examination on Tuesday, July 7, Wednesday, 8, and Thursday, 9, 1903, in the Assembly Room of the State-House at Trenton, N. J. Sessions will begin promptly at 9 A.M.

All applications must be in the hands of the secretary ten days prior to the examination.

J. Allen Osmun,
*Secretary Dental Commission.*

---

### ILLINOIS STATE DENTAL SOCIETY.

The thirty-ninth annual meeting of the Illinois State Dental Society will be held in Bloomington, May 12, 13, and 14, 1903. A large programme of interesting essays and clinics has been prepared, and a splendid meeting is expected.

The railroads throughout the State and from St. Louis will make a rate of a fare and one-third, certificate plan, for the round trip. All are cordially invited. Remember the date.

A. H. Peck, *President.*
Hart J. Goslee, *Secretary.*

### MISSISSIPPI BOARD OF DENTAL EXAMINERS.

THE Mississippi Board of Dental Examiners will meet in the city of Jackson, May 26, 1903, at 8.30 A.M.

J. P. BROADSTREET, D.D.S., *President.*
W. R. WRIGHT, D.D.S., *Secretary.*

---

### LEBANON VALLEY DENTAL ASSOCIATION.

THE twenty-eighth annual meeting of the Lebanon Valley Dental Association will be held at Harrisburg, May 19 and 20, 1903.

The programme, which is a very good one, will be forwarded in due time. *Come,* and bring something of interest with you.

A general invitation is hereby extended.

H. W. BOHN,
*Chairman Executive Committee.*

---

### KENTUCKY STATE DENTAL ASSOCIATION.

THE Kentucky State Dental Association has changed the date of meeting to May 25, 26, and 27, 1903, at Bowling Green.

C. R. SHATTUCK,
*Secretary.*

LOUISVILLE, KY.

---

### PENNSYLVANIA STATE DENTAL SOCIETY.

THE thirty-fifth annual meeting of the Pennsylvania State Dental Society will be held July 7, 8, and 9, 1903, at Harvey's Lake, Pa., which is delightfully situated near Wilkes-Barre.

This choice location is central, convenient to reach, and as near the ideal as is possible to obtain. Hotel accommodations are first-class and rates reasonable.

The Committee proposes to make this meeting a notable one; in fact, by far the best we have ever held in this State. An exceptionally interesting programme is being arranged, which includes papers and clinics from some of the most distinguished specialists.

Exhibitors have promised to lend their aid by exhibiting all the latest modern scientific devices. Every article of interest to the profession will be represented, and may be purchased from exhibitors. To keep posted regarding practical scientific advancements, this opportunity should appeal to you.

We ask every dentist, whether a member of the Society or not, to use his influence in making his own State meeting a memorable affair and a credit to the profession.

H. B. McFadden, D.D.S.,
Howard S. Seip, D.D.S.,
James P. Nichol, D.D.S.,
*Executive Committee.*

---

### MISSOURI STATE DENTAL ASSOCIATION.

The Thirty-ninth Annual Meeting of the Missouri State Dental Association will be held at Kansas City, Mo., May 19, 20, and 21, 1903.

Reduced railroad and hotel rates have been secured, and a very large attendance is assured.

A number of dentists of national reputation, among them Drs. J. B. Willmont, Toronto, Canada; E. K. Wedelstaedt, Minneapolis, Minn., and A. C. Searl, Owatonna, Minn., will present features and give clinics.

All ethical members of the profession are cordially invited to attend, become members, and take part in the discussions.

Otto J. Fruth,
*Corresponding Secretary.*

---

### NEW ORLEANS ACADEMY OF STOMATOLOGY.

The annual meeting of the New Orleans Academy of Stomatology was held in the assembly-room of the New Orleans College of Dentistry on Wednesday, March 11, 1903. After the transaction of routine business and the reading of interesting papers the following officers were elected to serve for the ensuing year:

President, Dr. H. P. Magruder; Vice-President, Dr. J. H. Landry; Secretary and Treasurer, Dr. Paul de Verges.

*Executive Committee.*—Dr. L. D. Archinard, Dr. C. V. Vignes, Dr. V. K. Irion.

The Academy meets on the fourth Wednesday of each month. The essayist at our next meeting will be Dr. M. R. Fisher.

PAUL DE VERGES,
*Secretary and Treasurer.*

### CENTRAL DENTAL ASSOCIATION OF NORTHERN NEW JERSEY.

AT the annual meeting of the Central Dental Association of Northern New Jersey, held at Newark, February 16, 1903, the following officers were elected:

President, William H. Pruden, D.D.S., Paterson; Vice-President, C. W. Hoblitzell, D.D.S., Jersey City; Treasurer, Charles A. Meeker, D.D.S., Newark; Secretary, Fred. W. Stevens, D.D.S., Newark.

*Executive Committee.* — J. A. Voorhees, D.D.S., Chairman, Newark; Frank Manning, D.D.S., Secretary, Elizabeth; J. S. Vinson, D.D.S., Newark; W. W. Hawke, D.D.S., Flemington; J. S. Dunning, D.D.S., Paterson.

FREDERICK W. STEVENS,
*Secretary.*

588 BROAD STREET, NEWARK, N. J.

### NEBRASKA STATE DENTAL SOCIETY.

THE Nebraska State Dental Meeting will be held in Lincoln, Nebraska, May 19 to 22, 1903, inclusive.

H. R. HATFIELD.

### MASSACHUSETTS DENTAL SOCIETY.

THE thirty-ninth annual meeting of the Massachusetts Dental Society will be held in Mechanics' Building, Boston, Wednesday and Thursday, June 3 and 4, 1903.

EDGAR O. KINSMAN,

*Secretary.*

CAMBRIDGE, MASS.

